# Continuous diffraction of molecules and disordered molecular crystals

**DOI:** 10.1107/S160057671700749X

**Published:** 2017-07-07

**Authors:** Henry N. Chapman, Oleksandr M. Yefanov, Kartik Ayyer, Thomas A. White, Anton Barty, Andrew Morgan, Valerio Mariani, Dominik Oberthuer, Kanupriya Pande

**Affiliations:** a Centre for Free-Electron Laser Science, DESY, 22607 Hamburg, Germany; b Department of Physics, University of Hamburg, 22761 Hamburg, Germany; c Centre for Ultrafast Imaging, 22607 Hamburg, Germany

**Keywords:** continuous diffraction, disordered crystals, statistics, photosystem II, diffuse scattering

## Abstract

The intensities of far-field diffraction patterns of orientationally aligned molecules obey Wilson statistics, whether those molecules are in isolation (giving rise to a continuous diffraction pattern) or arranged in a crystal (giving rise to Bragg peaks). Ensembles of molecules in several orientations, but uncorrelated in position, give rise to the incoherent sum of the diffraction from those objects, modifying the statistics in a similar way as crystal twinning modifies the distribution of Bragg intensities. This situation arises in the continuous diffraction of laser-aligned molecules or translationally disordered molecular crystals. This paper develops the analysis of the intensity statistics of such continuous diffraction to obtain parameters such as scaling, beam coherence and the number of contributing independent object orientations. When measured, continuous molecular diffraction is generally weak and accompanied by a background that far exceeds the strength of the signal. Instead of just relying upon the smallest measured intensities or their mean value to guide the subtraction of the background, it is shown how all measured values can be utilized to estimate the background, noise and signal, by employing a modified ‘noisy Wilson’ distribution that explicitly includes the background. Parameters relating to the background and signal quantities can be estimated from the moments of the measured intensities. The analysis method is demonstrated on previously published continuous diffraction data measured from crystals of photosystem II [Ayyer *et al.* (2016 ▸), *Nature*, **530**, 202–206].

## Abstract 2

The statistics of continuous diffraction patterns are determined and used to improve analysis of the diffraction of imperfect crystals of photosystem II.

## 1. Introduction   

**Figure 1:**
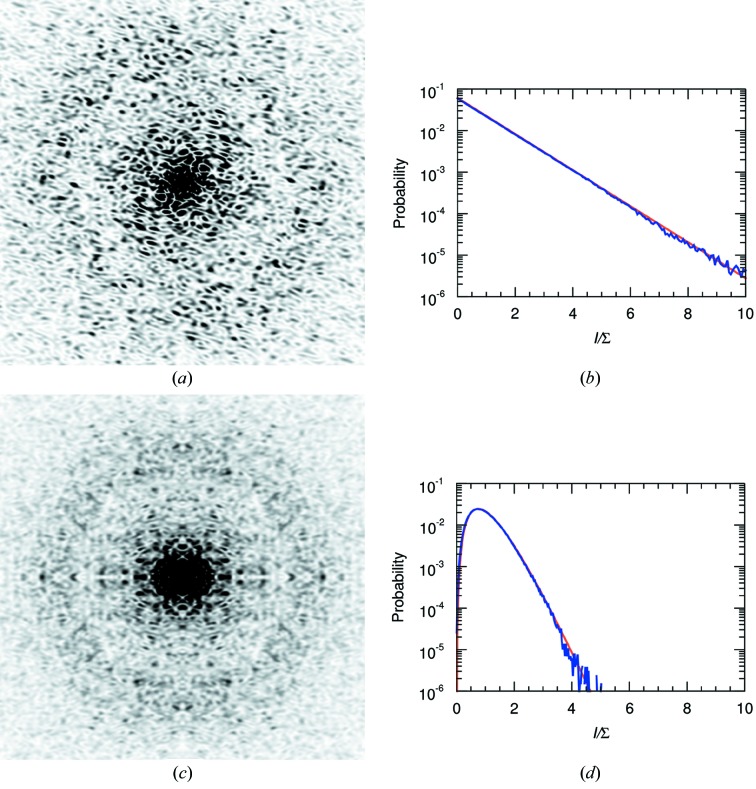
(*a*) A central section of diffraction intensities of PS II complex from a calculation at 475^3^ points in a three-dimensional array of reciprocal space, centred on the origin. The resolution at the centre edge of the simulated array is 0.33 Å^−1^ and intensity samples are spaced by 0.0014 Å^−1^, which is twice the Nyquist sampling of the intensities of an object 178 Å wide. (*b*) Histogram of intensity values at samples in a shell of reciprocal space of width 0.0325 Å^−1^ and centred at *q* = 0.23 Å^−1^ (blue), and the negative exponential of equation (1) in orange. (*c*) A central section from the same three-dimensional array after applying symmetry operations of the 222 point group, displayed on the same colour map as part (*a*), which ranges from zero counts in white to maximum counts in black. The section is only perpendicular to one twofold axis, which is horizontal in this view. Visually, the pattern has lower contrast than part (*a*), which is confirmed in (*d*) the histogram of intensity values which has a smaller width (*i.e.* smaller variance) for the same reciprocal-space shell as for part (*b*). The Gamma distribution *p*(*I*; 4) of equation (3) is given in orange.

The statistics of diffraction intensities in protein crystallography have guided data analysis and data verification, such as providing the basis for a treatment of negative diffraction intensities (French & Wilson, 1978 ▸), and the identification of crystal symmetries (Wilson, 1949 ▸; Rogers, 1950 ▸) and crystal twinning (Rees, 1980 ▸). The probability distribution of Bragg intensities in the X-ray diffraction pattern of a molecular crystal was first considered by Wilson (1949 ▸), now referred to in the field as Wilson statistics. The assumptions on which the derivation of these statistics depends, namely that the atoms in the molecule are random and independent, apply equally to the case of continuous coherent diffraction of a single mol­ecule (Huldt *et al.*, 2003 ▸). Such diffraction does not contain Bragg peaks since the object is not periodic, but is instead proportional to the squared modulus of the continuous Fourier transform of the molecule, such as the computed patterns shown in Fig. 1 ▸. These are also known as speckle patterns and are similar to the patterns that can be observed by shining an optical laser beam on a uniformly rough surface such as a painted wall. In both cases the contrast of the speckles is high and their size, which is roughly homogeneous, is inversely proportional to the size of the illuminated object (the diameter of the molecule or laser beam). This similarity holds for the statistical description of the intensities. Speckle patterns of laser beams reflected from rough surfaces also obey Wilson statistics and a body of literature, parallel to that of macromolecular crystallography, has presented derivations of intensity distributions and their experimental verifications, explored methods to reduce the contrast of speckle in cases where it is considered a nuisance, and utilized the statistics to determine coherence or roughness properties (Dainty, 1976 ▸; Goodman, 1985 ▸, 2007 ▸). Although optical speckle patterns were observed in the nineteenth century and explained by von Laue (1914 ▸) and Lord Rayleigh (1918 ▸), it was not until the invention of the laser that detailed examination was taken up. It is interesting that there was no such hindrance in X-ray crystallography, where X-ray sources provided beams that could be made coherent enough – using collimating devices – to give rise to coherent diffraction patterns (consisting of Bragg peaks obeying Wilson statistics) from molecular crystals.

With the more recent measurements and studies of continuous diffraction patterns of molecular samples, such as X-ray or electron diffraction of aligned molecules (Küpper *et al.*, 2014 ▸; Hensley *et al.*, 2012 ▸), single un-oriented molecules and viruses (Seibert *et al.*, 2011 ▸; Aquila *et al.*, 2015 ▸), or translationally disordered crystals (Ayyer *et al.*, 2016 ▸), an understanding of the distribution of continuous diffraction intensities is required for the same reasons as mentioned above for crystallography. The motivation for these studies is clear: the continuous diffraction, when sampled at or beyond its Nyquist frequency, provides a complete description of the diffracted wavefield and directly gives access to the full un-aliased autocorrelation function of the object. Under most conditions, the information content of the measurable diffraction intensities exceeds that required to describe the electron density of the sample completely, allowing iterative algorithms to retrieve the diffraction phases directly without the need for prior knowledge about the object, a method known as diffractive imaging (Thibault & Elser, 2010 ▸).

Perhaps the most crucial aspect of primary data analysis of continuous diffraction measurements is to determine the contribution of any incoherent background to the pattern. Unlike the narrow Bragg peaks in the diffraction patterns of crystals, which can be distinguished reasonably well from a slowly varying background (Parkhurst *et al.*, 2016 ▸), the morphology of a continuous speckle pattern makes this discrimination less straightforward. In addition, without the ‘coherency gain’ that concentrates intensity into Bragg peaks (Sayre & Chapman, 1995 ▸), continuous diffraction is much weaker per pixel than Bragg diffraction and any incoherent background may far surpass the strength of the signal of interest. As we shall see, the common assumption that local minima of the diffraction pattern should be zero and can thus be attributed to background is not always correct, especially when the particles are oriented in several discrete orientations, as can be the case for continuous diffraction of a translationally disordered crystal.

Indeed, the stimulus for this work was to better treat the continuous X-ray diffraction patterns that were measured from imperfect crystals of photosystem II (PS II) (Ayyer *et al.*, 2016 ▸). The presence of Bragg peaks in a diffraction pattern indicates a high degree of correlation of objects in a regular arrangement, over a number of objects that is inversely proportional to the width of the Bragg peak and to a precision determined by the highest scattering angles (the highest resolution) to which those Bragg peaks exist. Crystals of large protein complexes, such as membrane proteins, often only give Bragg diffraction to a limited resolution. The lack of correlation beyond this limit may be due to the objects being different from one another at this length scale, or to them being structurally alike but randomly displaced from the regular lattice. Combinations of these effects may also occur, including rotational disorder of rigid objects or translational disorder of conformationally varying molecules, but it is the lack of translational correlation that causes the Bragg peaks to vanish beyond a certain resolution. Scattering from the crystal still occurs beyond the highest-angle Bragg peaks, since the atomic scattering factors and numbers of atoms do not change just because the crystal is not ordered. In the case of translational disorder of repeating rigid units, the diminishing Bragg efficiency with increasing resolution is balanced by an increase in the incoherent sum of the continuous diffraction patterns of those units. This continuous diffraction is identical to that from a gas of molecules that are oriented in the discrete crystallographic orientations and is amenable to direct phasing using iterative methods, as demonstrated by Ayyer *et al.* (2016 ▸). It should be noted that continuous diffraction from molecular crystals is observed for a wide variety of systems and can take many general forms that can be more complicated than described by these two cases (Doucet & Benoit, 1987 ▸; Wall *et al.*, 1997 ▸; Pérez *et al.*, 1996 ▸; Van Benschoten *et al.*, 2016 ▸). Such ‘diffuse scattering’ is often of interest for the study of conformational variabilities of proteins. The correlations that give rise to such scattering can be anisotropic or localized in particular regions of reciprocal space (Welberry *et al.*, 2011 ▸). Such cases are not considered in this paper, although the concepts developed here might find some use to improve their analysis if properly adapted to the particular situation.

In this paper we review, in §2, the statistics of continuous speckle patterns of ensembles of molecules aligned in one or several discrete orientations for both centric and acentric structures. These statistics follow familiar distributions of Bragg intensities of twinned crystals, since the intensities arise in both cases from an incoherent sum of independent coherent diffraction patterns. As is also well known in the field of speckle metrology, this incoherent sum reduces the contrast of the pattern and increases the most common intensity value from zero. The consideration of coherence is more critical for continuous diffraction than for Bragg diffraction, since partial coherence reduces the contrast of speckle patterns and modifies their statistics, with consequences for the ability to phase them. We verify the predicted distribution of intensities of partially coherent diffraction patterns by simulation. Even when using a coherent source such as an X-ray free-electron laser (XFEL), the finite size of pixels in the diffraction detector gives the same effect, by reciprocity, as partial coherence.

In §3 we consider the statistics of the continuous diffraction of translationally disordered crystals or other collections of molecules in discrete orientations. While this continuous diffraction follows the point-group symmetry of the crystal, as does the Bragg diffraction, the distribution of intensities may be different from that of the Bragg intensities owing to the in­coherent addition of diffraction from rigid units, compared with the coherent addition of scattering from the entire contents of the unit cell that gives rise to Bragg diffraction. We consider some special central sections of reciprocal space that are perpendicular to the symmetry axes of the point group and which possess distributions that do not occur in diffraction of twinned crystals, and give some examples to highlight how the statistics of continuous diffraction could indicate or verify the symmetry of the rigid unit in a translationally disordered crystal.

We derive, in §4, the distributions of diffraction intensities consisting of the continuous diffraction of discretely oriented structures accompanied by an incoherent background. While the implications of subtracting background from Bragg data and the aforementioned treatment of negative intensities have long been considered (French & Wilson, 1978 ▸), with recent work improving the estimation of background in the neighbourhoods of Bragg peaks (Parkhurst *et al.*, 2016 ▸), there has not been a detailed investigation of the statistics of diffraction intensities in the case where the standard deviation of the background is a significant fraction of, or is larger than, the diffraction signal. We consider first the case where the background is normally distributed and give an explicit expression for the distribution of the intensities. Although we cannot obtain a similar expression for the case of photon-counting measurements, where the background follows a Poisson distribution, we determine the moments of intensities for both cases, and show that the parameters of the background (mean and variance in the case of a normal distribution) and the scaling of the signal can be solved from the moments of the measured intensities, given that the number of independent orientations of the diffracting structures is known. In §5 we apply this estimation of parameters in shells of reciprocal space to diffraction patterns measured from translationally disordered PS II crystals, as previously published (Ayyer *et al.*, 2016 ▸). In that work, the background was estimated simply from the average intensities in circular rings of constant *q*, but we show here improved results obtained by estimating the background level and diffraction signal scaling from the moments of the intensities. In §6, we examine the statistics of aggregated three-dimensional continuous diffraction from the scaled and background-corrected PS II patterns and find that the acentric intensities fit a distribution corresponding to the incoherent summation of four independent structures, consistent with the four crystallographic orientations of the PS II dimer. Finally, in §7 an improved cross correlation is observed between the background-corrected continuous diffraction and simulated diffraction from an atomic model of a PS II dimer.

## 2. Statistics of diffraction intensities of aligned molecules   

The distribution of intensities measured in the diffraction pattern of a molecular structure can be derived by considering the coordinates **x**_*i*_ of atoms in the object to be rationally independent or random (Schmueli & Weiss, 1995 ▸). Under those conditions, for a structure that is not centrosymmetric and for a large number of atoms with approximately equal atomic scattering factors, the contributions of atoms to the phases of the diffraction amplitudes, 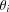 = 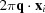, are uniformly distributed (between 0 and 2π) for any given photon momentum transfer **q**. The distribution of the magnitudes of the diffraction amplitudes, each a sum over the contributions from each atom, can then be estimated by analogy with a random walk in the complex plane or by application of the central limit theorem (Schmueli & Weiss, 1995 ▸; Dainty, 1976 ▸). Using the latter approach and for the case of unpolarized radiation, it is seen that, at a particular *q* (= |**q**|) shell (where atomic scattering factors do not vary), the real and imaginary parts of the complex-valued diffraction amplitudes are both normally distributed with a mean of zero and a mean square proportional to 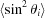 = 1/2 or 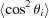 = 1/2. The diffraction intensities, *I*, are equal to the sum of the squares of the real and imaginary parts. The distribution of a sum of squares of *k* independent standard normal random variables is given by a χ^2^ distribution of order *k*, which can be scaled to any particular variance (Papoulis, 1991 ▸). Thus, the intensities *I* in a given shell of **q** are distributed according to a scaled χ^2^ distribution of order 2, with a probability distribution function given by 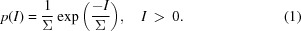
The mean of the intensity is Σ and it is set by the choice of the variance of the individual normal distributions. The variance of this distribution is Σ^2^ and the most common value (the mode) of *I* is zero. [This distribution is also referred to as a negative exponential distribution of scale Σ, an Erlang distribution with shape parameter 1 and rate 1/Σ, or a Gamma distribution with shape parameter 1 and scale Σ. In the notation of statistics, *I* ∼ Gamma(1, Σ), meaning that the random variable *I* has a probability distribution of Gamma(1, Σ).]

When the structure is real and centrosymmetric, then the phases of the diffraction amplitudes take on values of 0 or π, which is to say that the imaginary parts of the diffraction amplitudes are zero. This is also true for diffraction amplitudes on a central section (or zone) of reciprocal space perpendicular to any projection of the structure that is centrosymmetric, such as a projection along the twofold symmetry axis of a crystal. By the Fourier slice theorem, the Fourier transform of a real-space projection, an integration along a real-space direction of an object, is equal to the central section perpendicular to that direction of the three-dimensional transform of the object. The real parts of the diffraction amplitudes of the centrosymmetric object or projection will still follow a normal distribution, and thus the intensities, equal to their squares, will have a scaled χ^2^ distribution of order 1 (which can also be derived from the normal distribution by a change of variable), given by 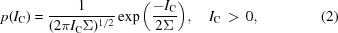
with a mean of Σ, a variance of 2Σ^2^ and a mode of zero. The intensities 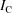 are referred to as centric.

Equations (1) and (2) are the well known Wilson statistics applicable to crystals of *P*1 symmetry and 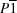 symmetry, respectively (Schmueli & Weiss, 1995 ▸), also referred to as Rayleigh statistics in the field of speckle metrology (Dainty, 1976 ▸; Goodman, 2007 ▸). The derivation of these statistics makes no assumption of crystallinity of the sample and hence they are as equally applicable to the continuous diffraction of a single object (Huldt *et al.*, 2003 ▸) [such as the calculated single-molecule diffraction of a PS II complex shown in Fig. 1 ▸(*a*)] as they are to the Bragg diffraction from a protein crystal, coherent diffraction from atomic glasses (Hruszkewycz *et al.*, 2012 ▸), or that resulting from the reflection of a monochromatic and polarized laser beam from a random rough surface (Dainty, 1976 ▸; Goodman, 2007 ▸). The applicability of Wilson statistics to single-molecule diffraction is demonstrated in Fig. 1 ▸(*b*), where the distribution of simulated point-sampled intensities in a shell of reciprocal space is plotted for the single PS II complex.

Equations (1) and (2) predict that the most common intensity value for molecular diffraction is zero. This is consistent with the view of a single-molecule diffraction pattern as made up of speckles that are surrounded by low values, such as seen in Fig. 1 ▸(*a*). The speckle nature of the diffraction is less easily observed in Bragg diffraction, but is certainly true given that the diffraction pattern of a crystal can be described as a modulation of the continuous diffraction of the unit cell with the reciprocal lattice. A difference from Bragg diffraction, however, is that single-molecule diffraction can be more readily affected by the spatial coherence of the illumination or, equivalently, the detector pixel-shape function, as discussed below in §2.3.

**Figure 2:**
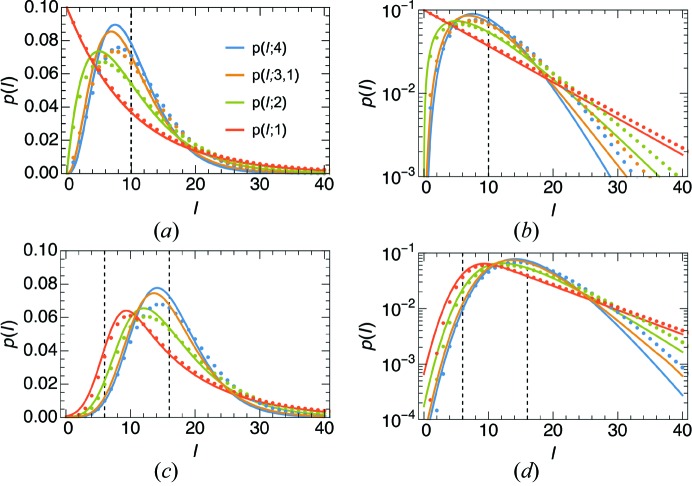
Plots of the distributions of diffraction intensities of disordered crystals with *P*2_1_2_1_2_1_ symmetry with a mean of Σ = 10 for continuously distributed values (lines) and for photon counting (dots). (*a*) Linear and (*b*) logarithmic plots of *p*(*I*; 4), *p*(*I*; 3, 1), *p*(*I*; 2) and *p*(*I*; 1), all without background, corresponding to the distributions for acentric continuous diffraction intensities, partly centric continuous diffraction intensities, centric continuous diffraction intensities (on central sections normal to crystallographic twofold axes) and acentric Bragg intensities, respectively. (*c*) Linear and (*d*) logarithmic plots of the ‘noisy Wilson’ distributions for the same cases with a background of μ = 6 and σ = 2.45. The dashed vertical lines correspond to the value of the mean signal, Σ, in parts (*a*) and (*b*), and the mean background, μ, and background plus signal, μ + Σ, in parts (*c*) and (*d*).

One insightful application of Wilson statistics is to identify the presence of crystal twinning purely from observations of diffraction intensities (Rees, 1980 ▸, 1982 ▸). The same tests can be carried out on diffraction of oriented single molecules. For example, alignment of molecules with an AC laser field gives rise to equal populations of molecules aligned parallel and anti-parallel to a laboratory-frame vector (Spence & Doak, 2004 ▸). As with the case of merohedral twinning of a crystal, the diffraction intensities of the two populations sum in­coherently. As long as the intensities at **R**·**q** are independent of those at **q** for the rotation operator **R** describing the twinning, then the distribution of the summed intensities follows the sum of two scaled χ^2^ distributions of order 2 (for a noncentrosymmetric object), which from the definition of a χ^2^ distribution is a χ^2^ distribution of order 4. In general, the diffraction intensity from *N* equal twin fractions (each with mean Σ/*N*) is given by a scaled χ^2^ distribution of order 2*N*, also equivalent to *I* ∼ Gamma(*N*, Σ/*N*), with a probability distribution function 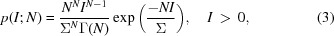
where Γ is the Euler Gamma function, equal to (*N* − 1)! for whole numbers of *N*. Some plots of *p*(*I*; *N*) are given in Fig. 2 ▸(*a*). The most common value for the continuous diffraction intensity for *N* 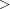 1 orientations is not zero but (*N* − 1)Σ/*N*. The mean of this distribution is Σ and the variance is reduced compared with the single object to a value of Σ^2^/*N* (see Table 1 ▸). The reduction in variance is quite noticeable in the simulated diffraction intensities shown in Fig. 1 ▸(*c*), which is the calculation of the incoherent sum of the diffraction of PS II complexes oriented in the four different orientations of the 222 space group (any orientation is related to another through a rotation of 180° about one of the three orthogonal axes). For the same mean, the standard deviation is halved in this case and the distribution of the simulated intensities agrees with equation (3) for *N* = 4, as seen in Fig. 1 ▸(*d*).

In the case of centrosymmetric objects in *N* unique orientations (whether due to crystal symmetry or twinning), the probability distribution will be given by the sum of random variables with scaled χ^2^ distributions of order 1, which is a scaled χ^2^ distribution of order *N*, *I* ∼ Gamma(*N*/2, 2Σ/*N*), with a probability distribution function 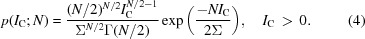
The distribution of equation (4) has mean Σ and variance 2Σ^2^/*N*, and is equal to *p*(*I*; *N*/2) when *N* is even. This will be the case for central sections of **q** that are perpendicular to the twofold rotation axis of a dimer, for example. In the limit of an infinite number of orientations, such as in the case of solution scattering of unoriented molecules, it can be found through the central limit theorem that *p*(*I*; *N*) and *p*(*I*_C_; *N*) both approach a normal distribution with a mean and variance both equal to Σ (Siegrist, 2015 ▸), which is also the limit of Poisson statistics.

We note that equations (1)–(4) [Disp-formula fd3] [Disp-formula fd4] hold for any scaling of intensities *I*, whether they be recorded as photon counts or as ‘detector units’, referred to herein as adu. For example, for a detector gain *a*, the intensities in detector units *I* = 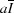 for the continuous diffraction of *N* orientations are distributed as *I* ∼ Gamma(*N*, *a*Σ/*N*), giving a probability distribution *p*(*I*) = 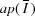 with a mean *a*Σ and variance *a* ^2^Σ^2^/*N*.

A case that breaks the independence between diffraction intensities at **R**·**q** and **q** is when the electron density of a single molecule is real valued, so that the diffraction intensities are centrosymmetric and the operator **R** is a rotation by 180°. Under this condition, the diffraction intensities *I*(**q**) of any object will be equal to *I*(**R**·**q**) for values of **q** in a central section that is perpendicular to the twofold rotation axis. In this reciprocal-space plane it would appear that the number of orientations of rigid objects is reduced by half or, equivalently, that the number of orientations does not change but the object’s projection is centrosymmetric. These diffraction intensities can thus be considered centric, even though the object itself is not centrosymmetric. Although similar to the case mentioned above of a crystal with a twofold rotation axis, there is a difference in that the projection of the structure of the crystal along the twofold axis is centrosymmetric, whereas it is the incoherent sum of the projections of aligned and anti-aligned molecules that is centrosymmetric. In the case of *N* equally populated alignment fractions, the centric reflections are those in central sections perpendicular to any twofold rotation axes in the point group of the alignments. In those planes, for a real-valued structure, the distribution of diffraction intensities will be given by equation (4) since the number of independent normal distributions being summed is reduced by half.

### 2.1. Discrete distribution   

Many of today’s X-ray detectors are sensitive to single photons, and diffraction measurements made with them are therefore governed by counting statistics. It is well appreciated that this discretization leads to a signal described by Poisson statistics. For example, the counts in a particular pixel on the detector in a diffraction experiment of a static object illuminated with a beam of constant flux will follow the probability distribution 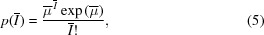
for a mean number of photons 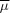, where 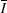 are the discrete numbers of photons per pixel (here the bar indicates values in photon counts). One feature of this distribution is that the variance is equal to the mean, 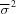 = 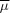. For large values of 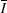 this distribution approaches a normal distribution with σ^2^ = μ. The statistics for the discrete diffraction of a molecule, measured at a particular *q* shell, are found by selecting a random variable from the appropriate Gamma distribution [*e.g.* equation (3)] and then realizing a particular value of that variable by feeding it as the mean value of a Poisson distribution. This is known as a mixture distribution and is conceptually quite different from the distribution of the sum of random variables discussed above. The mixture distribution of photon counts, where the Poisson mean is distributed according to 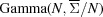 for *N* equal twin fractions, is given by the negative binomial distribution 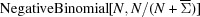,
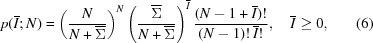
with a mean 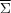 and variance 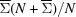 (ch 10.4 of Siegrist, 2015 ▸; Goodman, 2007 ▸). Thus, this distribution approaches the Poisson distribution for large *N* and the variance is greater than for the non-discrete distribution of equation (3). Some plots of the distributions are given in Figs. 2 ▸(*a*) and 2 ▸(*b*) for the case of 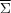 = 10 counts.

### 2.2. Linear polarization   

In the above we have assumed that the incident radiation is unpolarized. In that case, atomic scattering factors are dependent only on the magnitude of the photon momentum transfer *q*, giving rise to diffraction intensities that follow a given Gamma or negative binomial distribution for detector pixels located on a shell of constant *q*. Radiation at synchrotron and XFEL facilities is usually linearly polarized, which modifies the diffraction intensities by a factor equal to the square of the dot product of the electric field vectors of the incident and scattered rays (which themselves are perpendicular to the direction of propagation of the rays). For example, for horizontally (*x*) polarized radiation, the intensity pattern is modulated by 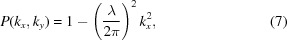
where 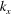 and 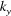 are the scattered wavevector components in the detector plane. The measured intensities of each diffraction pattern 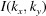 can be corrected by dividing by 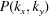. For measurements of a non-discrete diffraction signal, this correction will have the intended consequence of generating signals that follow the statistics of the Gamma distribution on a particular *q* shell. For photon-counting measurements this is not the case, since multiplying counts by a variable correction will alter the variance by a different factor than the mean. After correcting for polarization, counts in a *q* shell of the diffraction of an unstructured object will no longer obey Poisson statistics.

In our analysis below we utilize the variance to determine parameters such as the scaling and background of diffraction patterns. For discrete measurements we must first account for the polarization in this analysis, as follows.

First, the polarization-corrected diffraction pattern is averaged in thin shells of *q* (or *k*) from which a two-dimensional polarization-uncorrected average is regenerated: 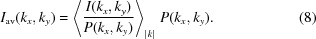
This average no longer contains any speckles but it can be contoured to find sets of detector pixels (or coordinates 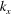, 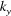) with equal mean counts Σ in the polarization-uncorrected measurement. These contoured regions are then used instead of shells of equal *q* to determine the distribution of intensities. This approach will account for any signal or background originating from elastic scattering from a region near the sample, but will not account for so-called detector dark noise, X-ray fluorescence, or scattering from sources far upstream or downstream of the sample.

### 2.3. Spatial coherence and pixel size   

**Figure 3:**
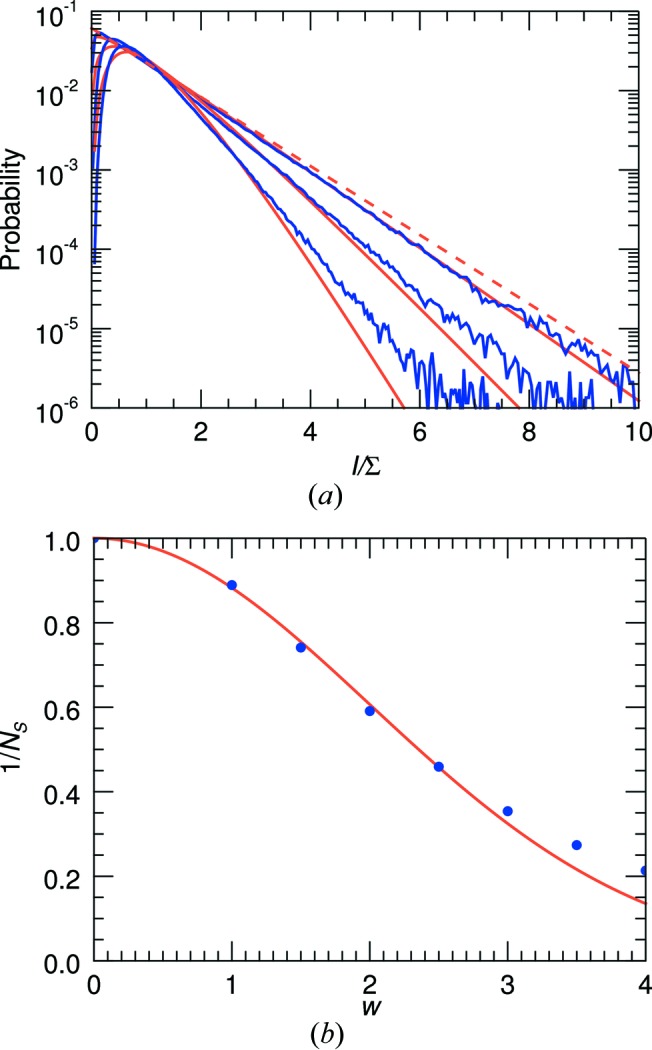
(*a*) Distributions of the simulated intensities of a single PS II complex after convoluting the three-dimensional reciprocal-space array of diffraction intensities with cubic voxels of widths 1, 2 and 3 times the Nyquist sampling rate of the continuous diffraction intensity. The negative exponential distribution of the point-sampled intensities is shown with the dashed line. (*b*) Plot of 1/*N*_S_ = Var[*I*]/Mean[*I*]^2^ *versus* the voxel width, *w*. The voxel width is normalized to the Nyquist sampling distance. Shown in orange is a Gaussian of width 2.

Continuous diffraction from single objects can be sampled arbitrarily finely, unlike the discrete locations of Bragg peaks. The simulated diffraction intensities shown in Fig. 1 ▸(*a*) were calculated at twice the Nyquist sampling rate required to fully describe the continuous intensity wavefield, which is to say four times the sampling density in each dimension that would be obtained from Bragg peaks of a *P*1 crystal in which the molecules were packed in the smallest possible *P*1 unit cell (see *e.g.* Thibault & Elser, 2010 ▸). At high sampling rates there are obviously correlations between neighbouring intensities, since they are likely to be sampling the same speckle. Even so, this ‘oversampling’ does not affect the statistics in the limit of randomly positioned atoms. The simulation does, however, differ from actual measurements of a diffraction pattern, in that intensities will not in reality be sampled at points but will be averaged over the active areas of the detector pixels, described by the convolution 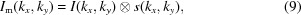
where 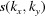 is the pixel response function. The statistics of the measurements *I*_m_ will clearly differ from those of *I* if the pixel is larger than the speckle size. The blurring by the pixel response reduces the contrast of the speckles, eliminating zeroes and raising low-intensity values as well as reducing the peak intensities. This can be seen in the plots of Fig. 3 ▸(*a*), where histograms of the simulated intensities of a PS II complex are given after first convoluting the patterns with cubic voxels of varying sizes. The distributions become more truncated and narrower, with an appearance similar to the distributions of the incoherent sum of *N* independent patterns as shown in Fig. 2 ▸.

The effect on the intensity statistics of a continuous speckle pattern convoluted with a pixel shape was examined by Dainty (1976 ▸), who showed that the variance of the intensities is reduced from the ideal value of Σ^2^ by a factor given by the ratio of the speckle size (equal to the inverse of the width of the autocorrelation function of the object) divided by the pixel area in *q* space. In particular, Dainty posited that the intensities *I*_m_ of equation (9) can be expressed as a weighted sum of independent random variables, and that the distribution can in fact be approximated (for a wide range of pixel response functions and molecule autocorrelation functions) by the Gamma distribution of equation (3). In this case *N* = *N*_S_, the number of speckles per pixel, need not be a whole number. In Fig. 3 ▸(*a*), these distributions are additionally plotted and can be compared with the histograms of the convoluted simulated diffraction patterns. The distributions show a good agreement with the simulations by setting 1/*N*_S_ = Var[*I*]/Mean[*I*]^2^. This parameter, referred to as the ‘speckle contrast’ (Dainty, 1976 ▸), can be considered as the degree of purity of the measurement in a detector pixel, or in other words an indicator of the degree of coherence, as discussed below. A plot of 1/*N*_S_ *versus* the voxel width of the recording process is given in Fig. 3 ▸(*b*), and 1/*N*_S_ is found to decrease with width roughly as a Gaussian. Here, the width is normalized to the Nyquist sampling width of the diffraction intensities (the inverse of twice the width of the molecule), which is about half the width of a speckle. The width of the Gaussian plotted in Fig. 3 ▸(*b*) is 2, equal to a speckle width.

The speckle contrast in a diffraction pattern can be used as a measure of coherence (Dainty, 1976 ▸). Reducing the spatial coherence of the illumination will reduce the variance of the diffracted intensities. This can be quite clearly understood in the Gauss–Schell model of partial coherence (Mandel & Wolf, 1995 ▸), where a partially coherent beam is equivalent to one produced by an incoherent source of finite extent. In this model, any point in the source gives rise to a fully coherent beam that produces a fully coherent diffraction pattern positioned relative to the axis defined by the line joining that source point and some arbitrary but common point in the object. For a small enough angular extent of the source, the pattern from each source point will be an identical but shifted version of that produced by any other source point. The patterns produced by each source point will be mutually incoherent, so in the limit of a small angular extent of the source the resulting diffraction pattern will be a convolution of the coherent pattern with a function describing the angular distribution of the source intensity. Thus, equation (9) also represents the case of partially coherent diffraction, where *s* describes the angular extent of the source, also equal to the Fourier transform of the mutual coherence function (Goodman, 1985 ▸). Again, this coherence length *w* can be expressed in terms of the parameter *N*_S_, equal to the fractional number of speckles that lie in the angular extent of the source.

In the case of *N* independent orientations of a molecule measured with *N*_S_ speckles per detector pixel or coherence area, the distribution will be modified in a similar way as for a single orientation. For a given mean Σ, the variance will be modified from Σ^2^/*N* by an additional division by *N*_S_ to Σ^2^/*N*′, where *N*′ = *NN*_S_, and the distribution of intensities will be approximated by *I* ∼ Gamma(*N*′, Σ/*N*′) as per equation (3).

The phasing of continuous diffraction patterns using iterative algorithms depends critically on accurate sampling of the intensities. Any reduction in contrast or addition of a constant will eliminate intensity zeroes and cause discontinuities of phased amplitudes, a situation that is inconsistent with diffraction arising from a compact object. Much progress in diffractive imaging was made recently by accounting for the decrease in contrast in continuous diffraction caused by partial coherence (Whitehead *et al.*, 2009 ▸). The coherence width, or equivalently the detector pixel width, is usually required as a fixed parameter in schemes of partially coherent diffractive imaging, and measurements of the coherence properties of the beam must often be made to carry out these schemes (Flewett *et al.*, 2009 ▸; Chen *et al.*, 2012 ▸). For macromolecular diffractive imaging, where the object is typically less than several hundred ångströms in width, achieving the necessary coherence width of the beam, equal to double the object width (Spence *et al.*, 2004 ▸), is routinely achieved, and the necessary sampling density and pixel width can be determined by examining the autocorrelation of the object. Nevertheless, the beam coherence, pixel width, sample heterogeneity and errors in aggregating data from many diffraction patterns may all give rise to an effective degree of coherence that can be determined directly from the intensity statistics if the number of object orientations is known. A variation in the determined coherence as a function of *q* may indicate rotational disorder of the molecules, or an alignment error in aggregating data from many single-molecule diffraction snapshots.

## 3. Statistics of diffraction intensities of translationally disordered crystals   

The diffraction pattern of a crystal exhibiting a degree of translational disorder consists of Bragg peaks, modulated by a *q*-dependent Debye–Waller factor, and continuous diffraction that arises contrariwise to the decrease in Bragg intensities. Ayyer *et al.* (2016 ▸) considered a disordered finite crystal consisting of a particular (and unique) rigid object that is repeated *M* times in different orientations and positions according to the crystal symmetry, in each of *K* unit cells of the crystal. Here a rigid unit is an object that can be considered as a single rigid body, such as the full photosystem II dimer in the case of the crystals in the study of Ayyer *et al.* (2016 ▸). The structures of these rigid units are identical, at least to the resolution considered in the diffraction pattern. The rigid bodies need not be an entire molecule or complex, however, and there may be several different rigid-body structures in the crystal. The three-dimensional diffraction pattern of such a crystal, with identical rigid units that are randomly displaced from their ideal crystallographic positions in each direction following a normal distribution of variance 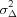, is then given by 
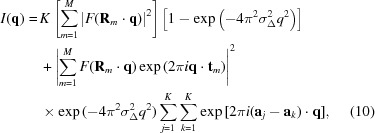
where *F*(**q**) is the complex-valued Fourier transform of the density of the single rigid unit, 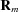 and 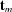 are the rotation and translation operators, respectively, for the *m*th rigid unit, and 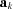 are the real-space lattice positions of the crystal. [Pre-factors in equation (10) are ignored.] Note that the isotropic mean-squared displacement of rigid units in three-dimensional space is equal to 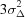. The second term of equation (10) is the squared modulus of the Fourier transform of the entire unit cell, modulated by a Debye–Waller factor and by the double product that gives the reciprocal lattice. The first term is markedly different and is given by the incoherent sum of the Fourier transforms of the rigid object in each of its crystallographic orientations, all modulated by a function, the complementary Debye–Waller factor, that increases monotonically with *q*. This term is not multiplied by a reciprocal lattice, and is thus continuous and proportional to the single-molecule diffraction when there is only one orientation of the rigid randomly translated object per unit cell. In the more general case, it is equal to the incoherent sum of the diffraction from *N* unique orientations of the rigid unit. The number of unique orientations of the rigid unit may be a subset of those given by the point group of the crystal if the rigid unit itself is crystallographically symmetric (*i.e.* not non-crystallographic), or there may be more orientations than dictated by the crystal symmetry if the rigid units are oriented according to non-crystallographic symmetry. An example of the former situation is given below for photosystem I crystals in the space group *P*6_3_.

The Bragg intensities are proportional to the coherent diffraction of the entire unit cell of the crystal [indicated by the squared modulus outside the sum in the second term of equation (10)] and hence depend on the space-group symmetry of the crystal. The continuous diffraction is proportional to the incoherent sum of the diffraction of the independent rigid objects in the crystal, subject to the (possibly reduced) point-group symmetry of the crystal given by the number of unique orientations of the (possibly symmetric) rigid objects. The statistics of the Bragg and continuous diffraction intensities are therefore different, depending on these symmetries. The Bragg reflections obey Wilson statistics with a symmetry dependence examined by Rogers (1950 ▸). The continuous diffraction will obey Wilson statistics such as given by equation (3) or (4), subject to the symmetry of the rigid unit and the number of unique orientations of that rigid unit. Equations (3) and (4) assume equal populations of objects in each of the orientations. In some crystals this will not be true, in which case the distributions can be derived from sums of squares of normally distributed random variables with different variances (Rees, 1982 ▸). In general, for a rigid unit with *N*_R_ non-crystallographic rotation operations and *N*_C_ crystal point-group operations, the centric intensities corresponding to any one of the non-crystallographic symmetries will be given by the incoherent sum of the centric diffraction from those objects in a particular orientation and the acentric diffraction from the rest of the objects. Assuming that the populations of rigid units in each crystallographic orientation are equal, these intensities will have a distribution *I* ∼ Gamma(*N*_C_ − 1, Σ/*N*_C_) + Gamma(1/2, 2Σ/*N*_C_). The probability distribution function of the sum of two random variables is equal to the convolution of their distributions, which can be calculated through the product of their inverse Fourier transforms. In statistics these are referred to as the characteristic functions (Papoulis, 1991 ▸; Schmueli & Weiss, 1995 ▸).

As an example, consider a PS II crystal in space group 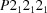. This consists of four dimers in unique orientations found by rotating any one of them by 180° about each of the three orthogonal axes of the orthorhombic cell. The twofold rotation symmetry of the dimer is non-crystallographic in this case, and the axis is not aligned along any of the crystallographic axes. The Bragg intensities are therefore in general acentric, given by equation (1). In central sections of reciprocal space perpendicular to the orthogonal crystal axes, however, the Bragg intensities are centric since projections of the crystal structure down those axes will be centrosymmetric, with a distribution given by equation (2). The projection of the crystal structure down the dimer twofold axis of any of the four dimers will not be centrosymmetric, however, since the crystal as a whole does not share this symmetry. The continuous diffraction of a PS II crystal with translational disorder will be governed by the incoherent sum of diffraction of equal populations of dimers in each of four orientations, given by equation (3) with *N* = 4, and will thus exhibit *mmm* symmetry. If the rigid unit is the dimer, then central sections perpendicular to the dimer twofold axis should include diffraction from the one-quarter of all dimers whose projections are centrosymmetric in that view. The statistics in that case will be determined by a sum given by three parts acentric random variables and one part centric random variables, resulting in *I* ∼ Gamma(3, Σ/4) + Gamma(1/2, Σ/2) which has the distribution 
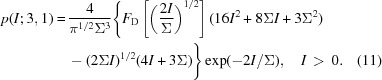
Here, *F*_D_ is the integral 
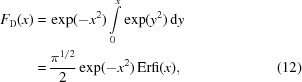
and Erfi is the imaginary error function, Erfi(*x*) = Erf(*ix*)/*i*. Plots of *p*(*I*; 4) and *p*(*I*; 3, 1) are given in Figs. 2 ▸(*a*) and 2 ▸(*b*), showing that the dimer symmetry causes a higher probability of high intensities compared with the completely acentric reflections. It should therefore be possible to detect the non-crystallographic symmetry of the rigid object from the deviations of the statistics in particular central sections of reciprocal space.

The central sections perpendicular to the three orthogonal crystal axes of PS II are all perpendicular to a twofold rotation axis and so, to the extent that the structure is real valued, these intensities will be centric, with a distribution given by equation (4) with *N* = 4. This is equal to the acentric distribution with *N* = 2, shown in Fig. 2 ▸.

As another example we consider a crystal of photosystem I, which has a hexagonal space group *P*6_3_. The structure consists of trimers with threefold rotational symmetry, located in alternating layers where the trimers are rotated by 60° about this threefold axis and translated perpendicular to it. The trimer symmetry is crystallographic. If the rigid object was hypothetically the entire trimer then the continuous diffraction arising from translational disorder would consist of the incoherent sum of the trimer in only these two orientations. Thus, in general, the probability distribution of the continuous diffraction intensities in any given *q* shell will be equal to *p*(*I*; 2) [equation (3) with *N* = 2]. The continuous diffraction of the trimer will have threefold rotational symmetry and, if the electron density of the trimer is real valued, will be centrosymmetric. In the central section perpendicular to the threefold axis the diffraction from the 60° rotated real-valued trimer will be identical, and hence in this plane of reciprocal space it will appear as if there is only one object contributing to the diffraction (or two centrosymmetric objects). These intensities can therefore be considered as centric, with a distribution in a given *q* shell equal to *p*(*I*; 1) [equation (3) with *N* = 1 or equation (4) with *N* = 2]. The Bragg reflections in general positions will be centric (*N* = 1), or acentric in the *hk*0 zone. Thus, comparisons of the statistics of Bragg and continuous diffraction in centric and acentric zones can, in principle, be used to constrain the number of rigid-body units contributing to the continuous diffraction. In practice, the contribution of noise to the measurement must be taken into account, since this modifies the intensity distributions, as discussed in the next section.

## 4. Modified statistics with background noise   

The continuous diffraction from a disordered crystal can be phased using iterative phasing algorithms, as has been well established for coherent diffractive imaging of single non-periodic objects. One of the experimental issues that can arise in coherent diffractive imaging is the incoherent addition of background intensity. For diffraction of disordered crystals, the diffuse scattering from the solvent adds to the pattern incoherently. This incoherent background must be estimated and subtracted, since otherwise phasing cannot be reliably achieved – the intensity sum does not match the squared modulus of the Fourier transform of an object of compact support. The complication can be appreciated by considering diffraction amplitudes that vary from positive to negative; for example, phases that vary from π to −π. The diffraction amplitude must therefore pass through zero, which cannot be satisfied if the measured intensity is everywhere greater than zero owing to a background.

When utilizing Bragg peaks alone, as is usual practice in crystallography, the background can be reliably estimated from the measured intensity values surrounding the peak. This obviously cannot be done for continuous diffraction. In that case, the background is often estimated from a measurement without the sample in place. In macromolecular crystallography, the sample is usually surrounded by solvent, which creates a diffuse background with a characteristic profile (including the so-called ‘water ring’). However, the crystal itself contains solvent which may differ in composition from the pure buffer solution, so the amount of background is not necessarily equal to the no-sample pattern. One way to estimate a smoothly varying background is to fit a function to the local minima of the diffraction pattern. This is a valid approach for an object of a single orientation whose distribution of diffraction intensities follows equation (1), but not when *N* 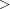 1. A much better approach is to utilize all intensity values in a reciprocal shell, not just the minima, and to fit the appropriate distribution to estimate the background. We explore this approach here by examining the properties of the distribution expected of intensities in a reciprocal-space shell from a disordered crystal with an incoherent background. This is carried out first for the case of non-discrete intensity measurements where the background in shells of *q* is considered to be normally distributed. This is the limiting case for large photon counts per pixel and allows analytical expressions of the resulting distribution of the sum of the aligned-molecule diffraction with the background. The case of discrete signals is presented in §4.2, where the background is assumed to follow Poisson statistics.

### 4.1. Non-discrete intensities with a normal-distributed background   

We refer to the distribution of the incoherent sum of the non-discrete acentric diffraction and a normally distributed background as the ‘noisy Wilson’ (NW) distribution. For a background mean μ and variance σ^2^, added to molecular diffraction of mean Σ from *N* orientations, the distribution is given by *p*_NW_(*I*) in equation (16) in Appendix *A* [App appa]. Some examples of the distribution are plotted in Figs. 2 ▸(*c*) and 2 ▸(*d*), where it is seen that *p*_NW_(*I*) is skewed. This skewness is a property of the signal following the Gamma distribution, rather than the skew-less normal-distributed noise. In situations of low signal to background, this skewness can therefore indicate the presence of a continuous diffraction signal. However, as we shall see in §4.2, unlike the normal distribution the Poisson distribution is skewed, with a skewness decreasing with the inverse of the square root of the mean counts. This is significant for mean counts approaching almost 100 photons, so the application of the results here requires suitably large signals or averages over many patterns.

As mentioned above, equation (16) can also be evaluated by the Fourier transform of the product of the characteristic functions of the Gamma and normal distributions. Likewise, it is possible to derive the moments of *p*_NW_(*I*) from the Fourier transform of the derivatives of its characteristic function. Such an analysis can also be carried out for the partially centric intensities that arise owing to non-crystallographic symmetry of the rigid unit, such as for the probability distribution *p*_NW_(*I*; *N* − 1, 1), even though an expression for the probability distribution cannot be readily derived. Expressions for these moments are given in Table 1 ▸ for the acentric and partially centric intensities. From the expressions in Table 1 ▸ it is possible to solve for the parameters Σ, μ and σ^2^ from the moments of the measured intensities in a given *q* shell. These parameters are, respectively, the mean of the continuous diffraction, the mean of the background and the variance of the background in that shell. This is far less computationally expensive than fitting a probability distribution function to those intensities to obtain the parameters. The solution to the simultaneous set of equations given by the first column of Table 1 ▸ yields the following expressions: 
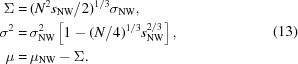
These estimates can be influenced by intensity values that do not conform to the expected distribution *p*_NW_(*I*), such as from Bragg peaks or centric intensities. The procedure of fitting the probability distribution function *p*_NW_(*I*) to the histogram of *I* tends to avoid the influence of outliers, but ideally any Bragg peaks should be identified and excluded from the analysis. The parameters Σ, μ and σ^2^ can be estimated in a number of reciprocal-space shells (*e.g.* 50 equally spaced shells) so that a smooth curve can be fitted to each of the parameters as a function of *q*. In this way a radially symmetric background μ(*q*) can be subtracted from the diffraction pattern. The curve Σ(*q*) can be used to generate a Wilson plot of the continuous diffraction (French & Wilson, 1978 ▸), the radially weighted average of which can be used to scale each pattern before merging with others to form a three-dimensional array of intensities. The error in the intensity measurements due to background can be estimated from σ(*q*).

### 4.2. Discrete intensities with a Poisson-distributed background   

The Poisson distribution is given by equation (5). The variance of this distribution is equal to the mean, 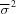 = 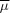, and the skew is equal to 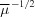, giving appreciable values of skew even for signals of tens of photons and showing that the analysis of §4.1 is suitable only in the limit of large photon counts. The distribution of the sum of acentric diffraction and unstructured background is given by 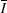 ∼ NegativeBinomial[*N*, *N*/(*N* + 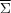)] + Poisson(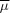). We refer to this as the ‘discrete noisy Wilson’ (DNW) distribution. An analytical expression for this distribution cannot be readily determined, but the probability distribution functions can be evaluated numerically using a program such as *Mathematica* (Wolfram Research, Champaign, Illinois, USA), as shown in Figs. 2 ▸(*c*) and 2 ▸(*d*). Additionally, the moments can be found from the characteristic functions of the distributions and these are given in the third column of Table 1 ▸ for intensities measured in photon counts. In this case, the expressions for the mean and variance are analogous to those for the normal-distributed background (replacing σ^2^ for 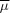) but the skewness differs in that it has a contribution from the background in the numerator. Unlike the case of the normal-distributed background, the skew is not a unique identifier of the presence of aligned-molecule diffraction. The mean acentric diffraction 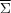 and mean background 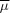 are determined by easily solving the two equations for 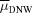 and 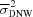 in Table 1 ▸: 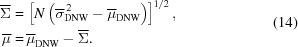
Compared with the continuous case, the presence of the signal is revealed by an excess of the variance of the intensities over the mean. Equality of these quantities would occur if the intensities followed a Poisson distribution, which would occur if no aligned-molecule diffraction signal were present. If 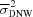 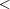 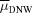 then the best estimate of 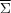 is zero.

The gain and offset of the detector can be estimated from a pattern recorded without any aligned-molecule diffraction but only a Poisson-distributed background, such as scattering from a liquid, or fluorescence. For a detector gain *a* and offset *b*, the mean intensity in detector units is 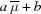 and variance 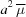. A linear fit to a plot of the sample variance as a function of the sample mean for different exposures or shells of *q*, for example, will give a slope equal the gain *a* and an offset *b*, assuming that the detector properties are the same over all pixels. For patterns recorded with linear incident polarization, the procedure outlined in §2.2 must be used to find groups of pixels in the polarization-uncorrected pattern from which to compute the mean and variance.

We note that, for an integrating detector, the noise model could be improved by adding a normal distribution corresponding to the detector noise. For the CSPAD detector (Philipp *et al.*, 2011 ▸; Hart *et al.*, 2012 ▸) used in the experiments described below, the standard deviation of the detector noise is below the signal for a single photon count, and thus only has a significant effect on the computation of statistics for patterns with very low detector counts. We ignore this consideration here.

## 5. Analysis of continuous diffraction patterns   

We demonstrate our analysis approaches on continuous diffraction patterns of PS II, previously measured at an XFEL (Ayyer *et al.*, 2016 ▸) by the method of serial femtosecond crystallography. Crystals in liquid suspension were jetted across the focus of the X-ray beam while snapshot patterns were recorded on every X-ray pulse (Chapman *et al.*, 2011 ▸; Boutet *et al.*, 2012 ▸) on a CSPAD detector. Measurements were carried out in a vacuum. The concentration of crystals in the jet was such that only a fraction of the pulses hit a crystal, and a set of diffraction patterns was selected by searching for the presence of Bragg peaks.

### 5.1. Statistics of a single pattern   

**Figure 4:**
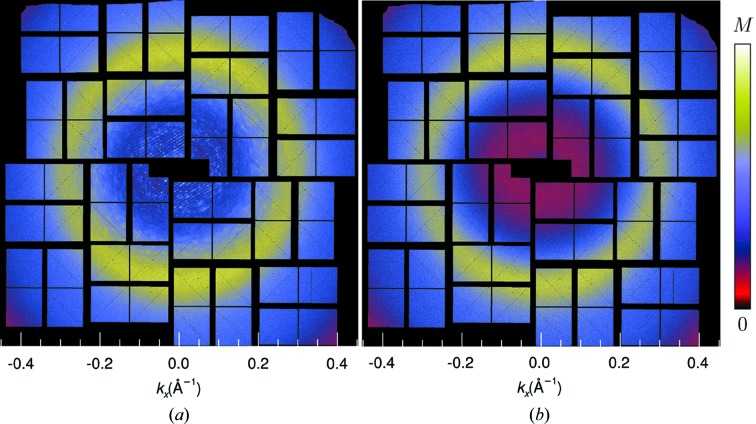
(*a*) Single-pulse FEL snapshot diffraction pattern of PS II, showing Bragg peaks and continuous diffraction from the disordered crystal and diffuse scattering from the solvent medium. (*b*) Single-pulse FEL pattern from the jet that was free of crystals, showing only the scattering from the liquid. The colour scale spans 0 to *M* = 3000 adu for part (*a*) and 0 to *M* = 2000 ▸ adu for part (*b*). The incident beam was linearly polarized, in the horizontal direction in this view. The patterns have not been corrected by the polarization factor.

A typical snapshot pattern (from a still, not rotating, crystal) is given in Fig. 4 ▸(*a*), without any background subtraction or correction for the polarization of the incident beam. The Bragg peaks obviously influence the statistics of the intensities and must be excluded from our analysis of the continuous diffraction. For this, they must first be identified, which was done by comparing the pattern with a version of itself that was modified by applying a median filter of width 9 pixels. A mask was defined by choosing pixels where the original values exceeded the median filtered values by an amount equal to the mean intensity value in the shell. This mask was then dilated using a kernel that was 7 pixels wide. While this was quite aggressive in removing regions around Bragg peaks, there were still a large number of pixels left to obtain histograms of the continuous diffraction intensities.

A pattern free of crystal diffraction and showing scattering from the liquid jet that carries the crystals is displayed in Fig. 4 ▸(*b*). Following the analysis procedure of §2.2, groups of pixels (excluding those that were masked) were determined by contouring 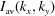 of equation (7) at levels spaced by 20 adu. A linear regression of the variances of the polarization-uncorrected intensities within these groups to the means showed a high degree of correlation (with a correlation coefficient of 0.998), giving a detector gain of 28.7 adu photon^−1^ and an offset of 29 adu.

**Figure 5:**
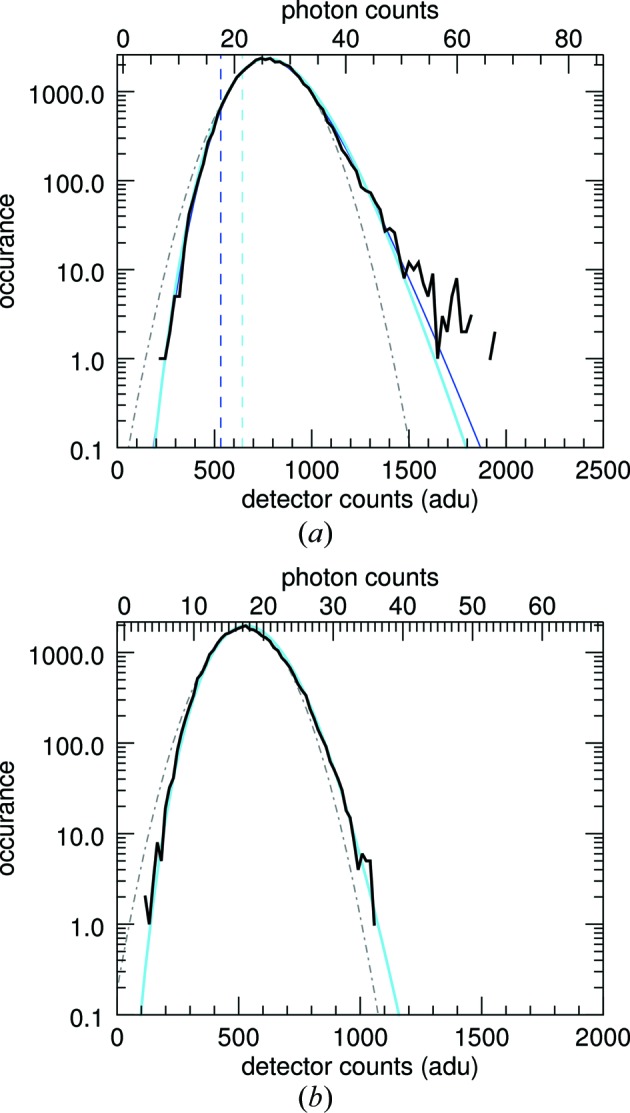
(*a*) Histogram (in black) of counts in a region of the pattern shown in Fig. 4 ▸(*a*), but prior to polarization correction, at a radius of approximately 260 pixels. The region was obtained by contouring the polarization-uncorrected radial average [see equation (8)]. A fit of *p*_NW_(*I*; 4) is shown in blue, yielding a value of μ = 532 adu = 18.6 photons, as indicated by the blue dashed line, and Σ = 269 adu = 9.4 photons. The discrete noisy Wilson distribution obtained by applying equations (14) is shown in sky blue and gives a higher estimate of the background with μ = 23 photons and Σ = 8 photons. (*b*) Histogram of counts in a region of the crystal-free pattern of Fig. 4 ▸(*b*), with a fit of a Poisson distribution (sky blue) with a mean of 18 photons. The dash–dotted grey lines indicate Gaussian fits, shown to emphasize the skewness of the distributions.

The distribution of intensities in a region of the pattern in Fig. 4 ▸(*a*) in a ring centred at about *q* = 0.15 Å^−1^ (260 pixel radius) is plotted in Fig. 5 ▸(*a*), in addition to the fits of *p*_NW_ and *p*_DNW_ with *N* = 4. The parameters obtained from the fit of *p*_NW_ were Σ = 269, μ = 532 and σ = 104 adu. Thus, the intensities are dominated by the background, as is obvious from Fig. 4 ▸(*a*). From the detector gain and offset determined above, these correspond to 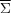 = 9.4, 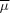 = 18.6 and 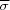 = 3.6 photons. The variance of the background 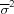 does not match the mean of the background, suggesting that the model of normal-distributed background does not well describe the data. By applying equations (14), the model of discrete statistics, to the same region after first converting the detector signal into photon counts, we obtain estimates of 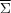 = 5.9 and 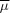 = 21 photons. That is, the discrete model yields a larger estimate for the background and a smaller estimate for the molecular diffraction. The non-discrete analysis determines the magnitude of the molecular diffraction signal based on the skew of the distribution, but photon counting creates an inherent skew in any case.

**Figure 6:**
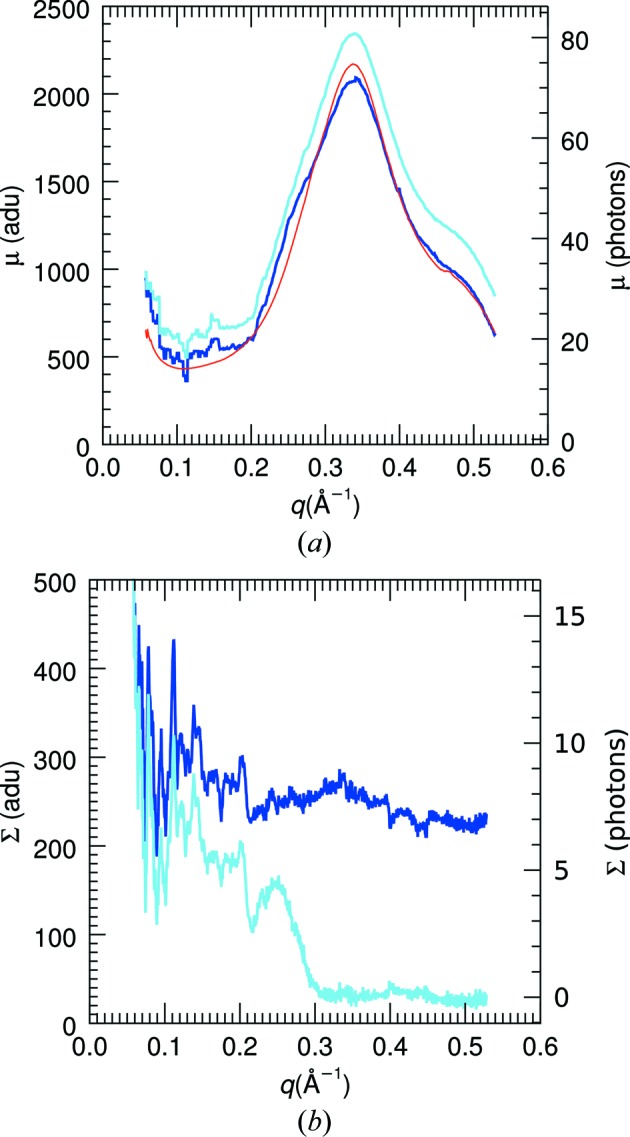
Plots of estimates of (*a*) the background and (*b*) the signal mean for the pattern of Fig. 4 ▸(*a*), obtained independently from contoured regions of the pattern obtained from the polarization-uncorrected radial average. Dark blue: μ(*q*) and Σ(*q*) obtained by applying equations (13) (non-discrete statistics) to the moments of the intensity values. Sky blue: μ(*q*) and Σ(*q*) obtained by applying equations (14) (discrete statistics) to the moments of the intensity values. Red: fitted radial average of a summed no-sample signal.

A plot of the estimated background μ, as a function of *q*, is given in Fig. 6 ▸(*a*) for the pattern of Fig. 4 ▸(*a*). The values obtained from the moments of the intensity values using equations (13), assuming non-continuous statistics, are plotted in blue, and those using equations (14) are plotted in sky blue. As with the values shown in Fig. 5 ▸(*a*) at *q* = 0.15 Å^−1^, the use of discrete distributions consistently estimates a higher background. The radial average of the no-sample background is also plotted (in red), scaled to fit the background estimates. The form of the background μ(*q*) matches the no-sample signal, but there are some differences which could possibly be due to a different composition of the solvent in the crystal compared to that in the buffer. Plots of the estimated signal Σ(*q*) are shown in Fig. 6 ▸(*b*). Here again, the estimate based on discrete statistics appears more reasonable, decreasing to nearly zero at the highest values of *q*. In that region the variance of the photon counts is approximately equal to the mean and hence is attributed to the Poisson-distributed background. We expect, from equation (10), that the continuous diffraction should be zero at *q* = 0 and modulated by 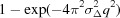. Nevertheless, even with this modulation which approaches unity at large *q*, the diffraction signal diminishes with *q* owing to its dependence on the atomic form factors and possibly to conformational variations in the molecules.

**Figure 7:**
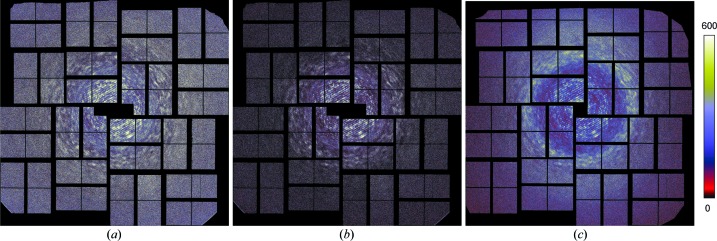
The pattern from Fig. 4 ▸(*a*) after subtracting the background μ(*k_x_*, *k_y_*) as calculated from the intensity moments for (*a*) non-discrete statistics and (*b*) discrete statistics, and (*c*) after subtracting the scaled no-sample signal. The colour scale ranges from 0 to 600 adu, corresponding to 0 to 23 photons.

Background-corrected patterns, obtained by subtracting backgrounds 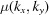 from the pattern of Fig. 4 ▸(*a*), are shown in Fig. 7 ▸ for the cases of non-discrete and discrete statistics. For discrete statistics, the background estimates were calculated in regions obtained by contouring the average pattern as calculated by equation (8), subtracting this map from the polarization-uncorrected pattern, before finally applying the polarization correction. In this way the variance in each region was calculated from the detected counts and was not affected by the polarization correction factor. Again, it is clear that the application of discrete statistics gives a more reasonable result.

The total photon count of the background-corrected pattern shown in Fig. 7 ▸(*b*) is 2.7 × 10^6^ photons, which is only 2.6% of the total count before background subtraction which is 1.03 × 10^8^ photons. Furthermore, the Bragg peaks account for 0.58 × 10^6^ photons. This was found by summing the values in pixels defined by the dilated mask mentioned above, which generously encompasses all Bragg peaks and thus could be considered an overestimate. It may be somewhat surprising that the continuous diffraction contains about 4.6 times the number of photons contributing to Bragg peaks. The total scattering power of the asymmetric units does not change, regardless of whether those units are arranged in a strictly periodic fashion or not, as can be seen from equation (10). The continuous diffraction extends over a much larger area of reciprocal space, which may account for the factor of 4.6. However, the atomic scattering factors are stronger at low *q* and so one may expect a greater proportion of the total scattering in the Bragg peaks, depending on how many data are missing at lowest *q*. Nevertheless, it is clear that the continuous diffraction is not weaker in total than the Bragg diffraction. Since it is not concentrated into narrow Bragg peaks but spread over many pixels of the detector, the signal-to-noise ratio of the continuous diffraction is lower than that of the Bragg data. From Figs. 6 ▸(*a*) and 6 ▸(*b*) the noise, given by 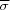 = 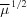, is comparable to the signal. Note, however, that individual speckles cover more than 100 pixels, so these are measured with a higher signal-to-noise ratio.

### 5.2. Statistics of an ensemble of patterns   

**Figure 8:**
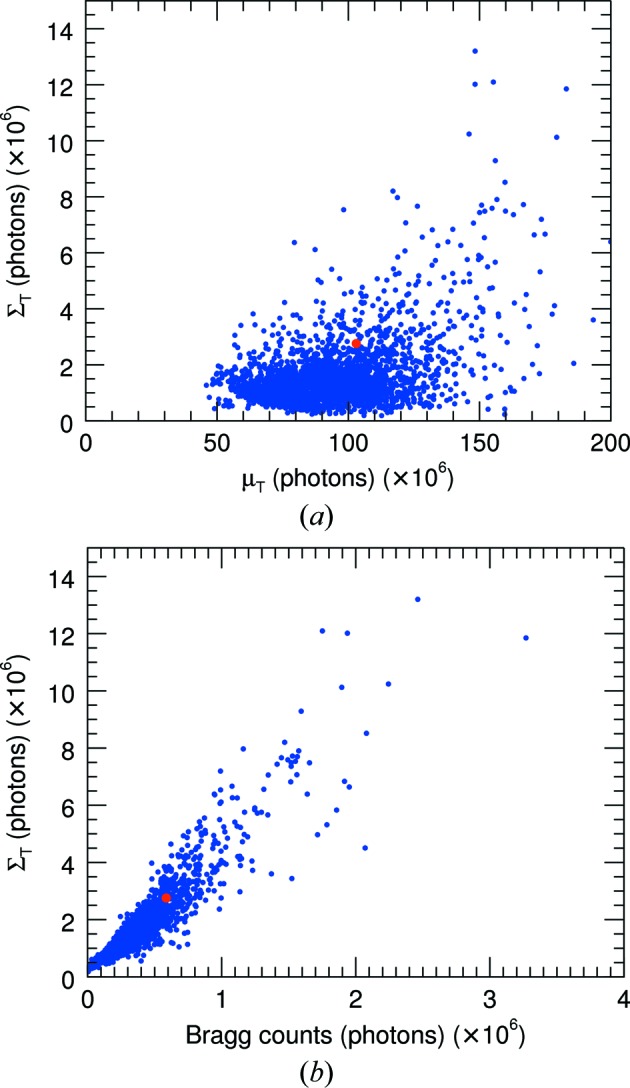
Plots of the total continuous diffraction signal strength, 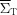, (*a*) as a function of the total background strength 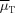 and (*b*) as a function of the total Bragg counts. The continuous diffraction signal is correlated with Bragg counts but not with the background. The continuous diffraction signal of the strongest patterns is about 5–10% of the background and more than four times the strength of the Bragg signal. The red points indicate the pattern shown in Fig. 4 ▸(*a*).

The analyses described in §5.1 can be repeated on the set of snapshot diffraction patterns recorded in a serial crystallography experiment, in order to obtain statistical measures of the experiment or to guide strategies for combining patterns into a data set (see §6). The PS II data set reported by Ayyer *et al.* (2016 ▸) consisted of 25 585 snapshot patterns with Bragg peaks that could be indexed to obtain the orientation of the diffraction in the frame of reference of the crystal lattice. In that work, the strongest 2848 patterns were oriented and aggregated in a three-dimensional reciprocal-space array for phasing. That subset was re-analysed to obtain parameters 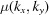 and 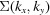 in regions of near-constant photon counts in the polarization-uncorrected patterns. The overall strengths of the background 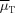 and the continuous diffraction signal 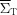 in the PS II patterns was estimated for each pattern by summing the parameters over the entire pattern, weighted by the areas of each region. In Fig. 8 ▸(*a*) the dependence of the total signal 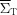 is plotted as a function of the background 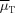. The strength of the diffraction is about 1–5% of the background. It is not strongly correlated with the background except that the very strongest diffraction signals coincide with the very strongest background. This trend may suggest that a portion of the background is inherent to the liquid jet, with higher pulse energies giving rise to both strong background and strong diffraction. Atomic diffuse scattering caused by disorder of the atoms in the molecules may also contribute to some portion of the background, perhaps induced by the pulse itself and building up during the course of the pulse (Barty *et al.*, 2012 ▸). The pattern shown in Fig. 4 ▸(*a*) is indicated by the red dots in Fig. 8 ▸, with typical signal and background strengths.

A plot of the continuous diffraction signal as a function of the total Bragg counts is given in Fig. 8 ▸(*b*), indicating a high degree of correlation. As with the pattern discussed in §5.1, the continuous diffraction strength is about four times that of the Bragg counts, on average. The plot suggests that the strength of the continuous diffraction depends on the volume of the crystal in the same way that the total Bragg count depends on the total number of unit cells contributing. This strong degree of correlation also indicates that all crystals possess a similar degree of disorder, such that the fraction of scattered counts in Bragg peaks *versus* continuous diffraction is roughly constant.

## 6. Statistics of the three-dimensional continuous diffraction intensities   

A three-dimensional data set of the continuously varying diffraction intensities of PS II was constructed using the approach described by Yefanov *et al.* (2014 ▸) and Ayyer *et al.* (2016 ▸). Briefly, in this approach the orientation of each snapshot diffraction pattern was determined by indexing its Bragg spots using the software *CrystFEL* (White *et al.*, 2012 ▸, 2016 ▸). The pattern was then interpolated onto the appropriate spherical surface (the Ewald sphere) in a three-dimensional array of reciprocal space, where the coordinates of the array were chosen to be parallel to the reciprocal-lattice axes. Compared with the previous work (Ayyer *et al.*, 2016 ▸), the smoothed background μ(*q*), interpolated onto the detector plane, was first subtracted from each pattern, which was also scaled by 1/Σ_T_, before merging into the three-dimensional volume.

**Figure 9:**
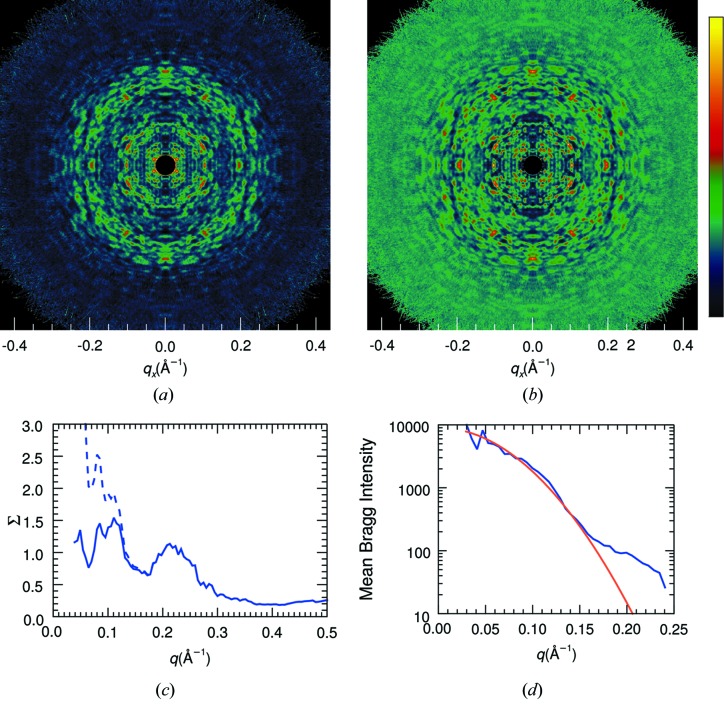
(*a*) A central section of the merged volume of continuous diffraction intensities using the method of this paper, and (*b*) the previously published results [reproduced with permision from Ayyer *et al.* (2016 ▸), copyright (2016) Nature Publishing Group]. The central sections are normal to [101], chosen to avoid the centric planes. The colour scale is indicated to the right and varies from −0.6 to 6 for part (*a*) and −100 to 250 for part (*b*). (*c*) The scaling Σ(*q*) obtained by fitting the distribution *p*_NW_(*I*; 4) to the merged continuous diffraction intensities in three-dimensional shells of *q*. The dashed line gives the scaling corrected for the complementary Debye–Waller factor with σ_Δ_ = 2.01 Å. (*d*) The scaling Σ(*q*) obtained by fitting *p*_NW_(*I*, 1) to merged Bragg intensities in three-dimensional shells of *q* in blue and the fit to a Debye–Waller factor with σ_Δ_ = 2.01 Å in orange.

The 2514 strongest patterns were chosen based on the values of Σ_T_. After merging the patterns into a three-dimensional array, the symmetry operations of the point group 222 were then applied, corresponding to summing the three-dimensional intensity array with copies of itself rotated about each of the three orthogonal axes of the crystal. This symmetrization simply averages equivalent observations of intensities in order to increase the signal-to-noise ratio. There is no loss of information in carrying out these operations since the crystal exhibits this symmetry anyway and the averaging cannot be avoided. We could also choose to impose centrosymmetry, which loses any information pertaining to Bijvoet differences. A map of the merged intensities in a central section normal to the [101] axis of the crystal is given in Fig. 9 ▸(*a*), which can be compared with the previously published results in Fig. 9 ▸(*b*) that were obtained by subtracting the radially averaged intensity from each pattern (Ayyer *et al.*, 2016 ▸). Fig. 9 ▸(*b*) appears to show more detail and contrast at high resolution, at least with the chosen colour scale. This may not be surprising, given that the no-crystal patterns were typically fitted before subtraction, resulting in high contrast and negative intensities, neither of which accurately represents the incoherent sum of molecules in several orientations. The new method avoids over-subtraction of background and provides an improved scaling of the patterns.

**Figure 10:**
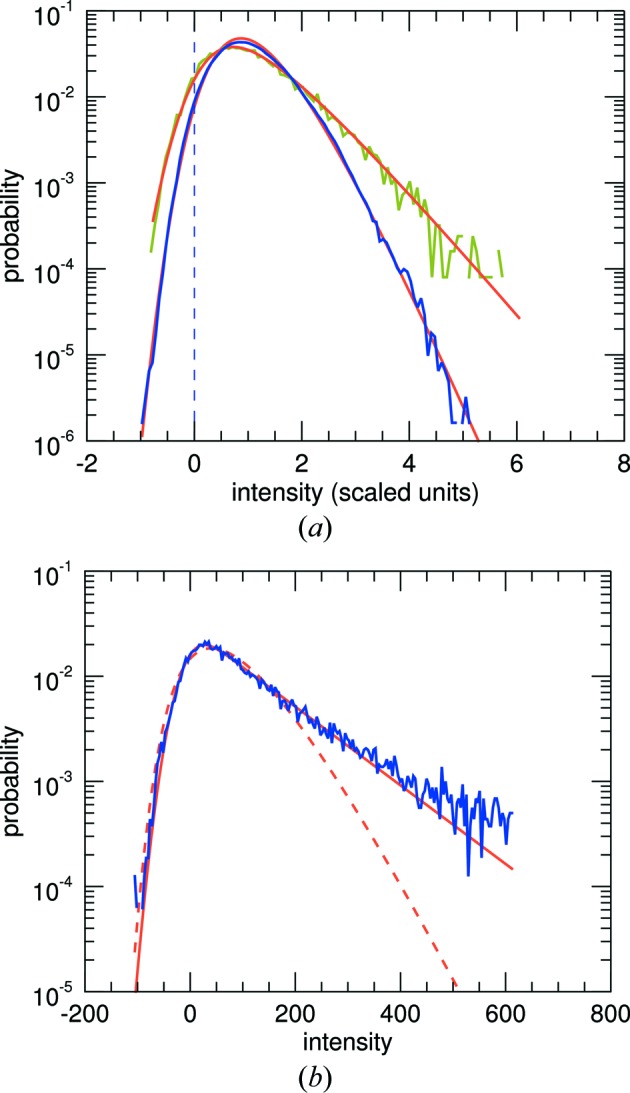
(*a*) Normalized histograms of intensities in the three-dimensional continuous diffraction for voxels excluding central sections parallel to each of the twofold rotation axes (blue) and for voxels lying on those planes (green), all contained within a shell 0.213 < *q* < 0.231 Å^−1^. The orange lines are fits of *p*_NW_(*I*; 4) and *p*_NW_(*I*; 2). (*b*) Histogram of Bragg intensities in a shell 1/6 < *q* < 1/5 Å^−1^ and excluding centric reflections (blue), with a fit of *p*_NW_(*I*; 1) shown in orange. The orange dashed line is a fit of *p*_NW_(*I*; 4), showing that the Bragg intensities do not result from twinning.

The statistics of the diffraction intensities in the three-dimensional volume can be used to verify the scaling and placement of the data and to verify the number of independent orientations of the rigid objects. For PS II crystals, which have 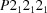 symmetry, we expect that the non-centric continuous diffraction follows the distribution *p*_NW_ of equation (16) with a twinning of *N* = 4 and that the zones perpendicular to each of the crystallographic twofold axes will be twinned with *N* = 2. The intensities are expected to follow the non-discrete Gamma distribution with a normally distributed background since they arise from the sum of many scaled (and background subtracted) patterns. Histograms of intensities chosen from the shell lying between voxel radii of 170 and 185 (0.213 < *q* < 0.231 Å^−1^) are given in Fig. 10 ▸(*a*), excluding the volume within 10 voxels of the three orthogonal zones, and for only those voxels lying on the three orthogonal zones. The histograms are normalized to total unity, giving an experimental probability distribution. It is immediately seen that the two distributions are indeed different, and fits of equation (16) can be obtained for *N* = 4 and *N* = 2, respectively (orange lines). Furthermore, the fits were obtained for almost the same diffraction intensity mean, Σ, as expected. The fitted parameters (in arbitrary units owing to the scaling) were σ = 0.30 and Σ = 1.05 for the *N* = 4 ‘non-centric’ intensities, and σ = 0.36 and Σ = 1.10 for the *N* = 2 centric intensities. Although the residual background level given by μ was low, it was subtracted to set this to zero. Thus, there are some remaining negative intensities due to the distribution of the noise. The average per-voxel signal-to-noise ratio of the three-dimensional intensities in this shell is Σ/σ = 3.5, larger than that of the individual patterns owing to signal averaging. With 2514 patterns included in this merged data set, the multiplicity at *q* = 0.22 Å^−1^ was 24. The larger standard deviation for the centric intensities than for the non-centric intensities can be attributed to the smaller sample size (5.0 × 10^4^ *versus* 4.9 × 10^6^ voxels).

The voxels of the three-dimensional array have a width of 0.00125 Å^−1^, which is larger than the width of the detector pixels. The largest diameter of the PS II dimer is 178 Å, and thus the largest extent of its autocorrelation is 356 Å. The spacing for Nyquist sampling of the diffraction intensities, which are equal to the Fourier transform of the intensity pattern, is thus 1/356 Å^−1^ = 0.0028 Å^−1^, giving a voxel width relative to this of *w* = 0.45. From Fig. 3 ▸(*b*) this should not have an impact on the coherence of the merged pattern or the intensity statistics.

The Bragg intensities obtained by processing all 25 585 diffraction patterns using *CrystFEL* are found to follow a negative exponential distribution, as shown in Fig. 10 ▸(*b*) for a shell with 1/6 < *q* < 1/5 Å^−1^, excluding centric reflections. For this shell the intensities could be fitted to the noisy Wilson distribution *p*_NW_(*I*; 1) with a mean signal Σ = 116 units, a background μ = −10.3 units and a standard deviation σ = 27.5. The expected distribution in this case is for *N* = 1, since there is no ambiguity of crystal orientation due to merohedry and hence no effective twinning. The intensities arise from coherent diffraction of the entire unit cell, and there is only one instance of that unit cell contributing to the Bragg intensities. The signal-to-noise ratio in this shell is Σ/σ = 4.2, which is only moderately greater than for continuous diffraction, even though ten times the number of patterns contribute and the intensities are concentrated into Bragg peaks. The distribution of Bragg intensities is clearly different from the continuous diffraction, as can be seen in the slopes of the distributions in Fig. 10 ▸. Other values of *N* do not fit as well to the distributions of continuous and Bragg intensities. The fitting essentially amounts to the early twin tests (Rees, 1980 ▸), which identified twinning by comparing the form of the cumulative distribution of intensities to the appropriate Gamma distributions. The fits here confirm that the continuous diffraction is indeed due to the incoherent sum of four independent objects, whereas the Bragg diffraction arises from a single untwinned crystal. This is supporting evidence that the continuous diffraction measured from the PS II crystals does indeed arise from translational disorder of PS II dimers and not the monomers, since there are four orientations of PS II dimers in the crystal but eight orientations of monomers. The statistics alone can not reveal if the four objects are identical, but the symmetry of the continuous diffraction suggests that, if they are different, then they are equally distributed over the four orientations. The statistics also suggest that the background-corrected continuous diffraction does not have a significant contribution due to structural variability such as conformational disorder, since diffraction from many smaller sub-structures would give rise to intensities approaching a Poisson distribution (the large-*N* limit of a Gamma distribution) and such diffraction would presumably not be completely rotationally invariant as was the Poisson-distributed background that was subtracted from each pattern. Some degree of orientational disorder of the rigid units is certainly possible, which would have the effect of reducing the diffraction contrast with increasing *q*, due to the blurring of speckles, as discussed in §2.

The scaling of the Bragg intensities as a function of *q* is shown in Fig. 9 ▸(*d*), plotted on a log scale, for comparison with the continuous diffraction plotted in Fig. 9 ▸(*c*) on a linear graph. This scaling predominantly follows the familiar Wilson plot of Bragg intensities and the Debye–Waller factor 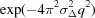 was fitted with σ_Δ_ = 2.01 Å, which can be equated with an overall *B* = 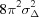 = 320 Å^2^. That is, this is the *B* factor computed by attributing the reduction in Bragg intensity with *q* to atomic displacement, whereas it is clear from the existence of continuous diffraction that the variation in Bragg intensities with *q* is mainly due to rigid-body displacements of the molecular complexes. The effect of the complementary Debye–Waller factor 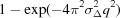 on the continuous diffraction is to suppress intensity at values of *q* 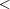 0.1 Å^−1^. At higher photon momentum transfer than this, the factor is greater than 0.8 and thus has little effect. The mean intensity of the continuous diffraction, corrected for this factor, is given in Fig. 9 ▸(*c*) as the dashed line.

## 7. Comparison with atomic model   

**Figure 11:**
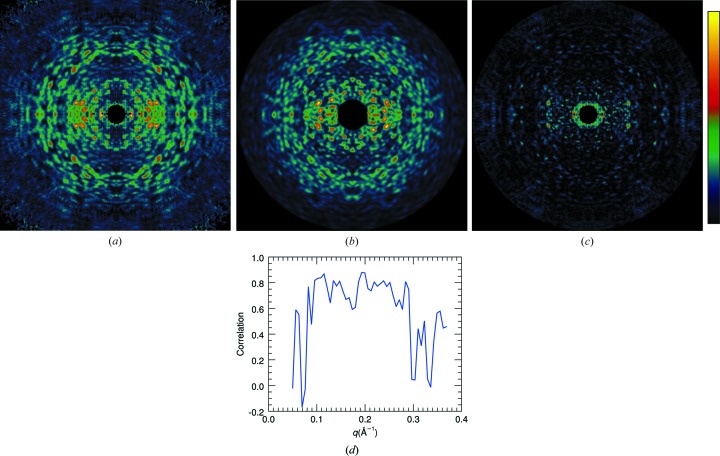
(*a*) A central section of the merged volume of continuous diffraction intensities, normal to the [010] lattice vector, compared with (*b*) the same section of the simulated continuous diffraction assuming a rotational disorder of 1° r.m.s. and a translational disorder of σ_Δ_ = 2.01 Å. (*c*) The difference between the experimental and simulated intensities, shown on the same colour scale as parts (*a*) and (*b*). (*d*) Plot of the Pearson correlation in shells of *q* between the experimental and simulated data.

As a final analysis of continuous diffraction, we compare it with the simulated continuous diffraction of a disordered crystal of PS II as calculated from an atomic model. For the model we used atomic coordinates obtained by a refinement of a structure of the PS II dimer to the electron density obtained by diffractive imaging (Ayyer *et al.*, 2016 ▸). The molecular transform *F*(**q**) of the PS II dimer was calculated by summing diffracted waves scattered from each atom on a three-dimensional array of **q** vectors spaced by 0.0025 Å^−1^ (twice that of the merged experimental data). From this the squared modulus |*F*(**q**)|^2^ was calculated, before applying the rotation operations 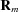 of the point group of the crystal and in­coherently summing the four equally weighted sets of intensities. The three-dimensional array was then multiplied by a factor 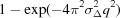 using the previously determined value of σ_Δ_ = 2.01 Å. It was found that the Pearson correlation between the experimental and computed data for the volume within the shell 0.088 < *q* < 0.29 Å^−1^ was 0.67, compared with a value of 0.55 obtained previously (Ayyer *et al.*, 2016 ▸). An even higher degree of correlation of 0.77 was obtained by blurring the computed intensities slightly by assuming rotational disorder of the PS II dimers by 1° r.m.s. To simulate this disorder, the symmetrized intensities were rotated in all three directions by amounts chosen from a normal distribution with a width of 1°. This was repeated 500 times and the results averaged. Fig. 11 ▸ displays the experimental and calculated intensities, this time on one of the centric zones (normal to the 010 lattice vector), and the difference, all on the same colour scale. No manipulation of the background or scaling of the data was made – that is, there are no fitted parameters other than the 1° rotational blurring. A plot of the Pearson correlation coefficient computed in shells of *q* is also given in Fig. 11 ▸. This reaches a maximum value of 0.88. A very similar result was achieved by uniformly convoluting the computed data by a 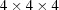
 voxel kernel instead of applying rotational blurring. The high degree of correlation confirms the origin of continuous diffraction and validates the approach of distinguishing molecular diffraction from structureless background.

## 8. Discussion and conclusions   

We have carried out an extensive analysis of the continuous diffraction arising from translationally disordered crystals of PS II that was used previously for macromolecular coherent diffractive imaging (Ayyer *et al.*, 2016 ▸). That the diffraction could be directly phased and used to obtain a volume image of the electron density of the PS II dimer was certainly strong evidence that the continuous diffraction originates from the incoherent sum of randomly displaced rigid objects (the PS II dimers), but here that particular analysis was expanded with rigorous statistical tests to gain a deeper understanding of the nature of continuous diffraction and how to measure it. One of the most crucial aspects in treating continuous diffraction is distinguishing it from diffuse (*i.e.* structureless) background scattering. Unlike Bragg peaks, which can easily be distinguished from slowly varying background in a diffraction pattern, continuous diffraction cannot be readily separated from such background. In the previous work (Ayyer *et al.*, 2016 ▸), the background was simply estimated from the radial average of the patterns. Such background was fitted to each crystal diffraction pattern, with the result of maximizing the contrast of the speckles of the molecular diffraction that ultimately led to an over-subtraction and negative intensities. In this work the statistics of the molecular diffraction intensities were exploited to obtain estimates of their scaling and zero level. In particular, the intensities in a shell of reciprocal space are assumed to follow a ‘noisy Wilson’ distribution, which is the distribution due to the sum of random variables describing the structured signal and the unstructured background, where the signal follows the familiar Gamma distribution of Wilson statistics and the noise follows a normal distribution. When photon counting is considered, this corresponds to the sum of discrete random variables from a negative binomial distribution and a Poisson distribution.

The statistics of the structured component of continuous diffraction depend on the number of independent objects contributing, or the number of modes in the speckle pattern. There are four orientations of dimers in PS II crystals and, since the displacements of each are random and uncorrelated, the diffraction from each orientation adds incoherently, giving rise to continous diffraction with the same point-group symmetry as the Bragg intensities. The statistics of the intensities thus do not follow the usual negative exponential of a single object, where the most common intensity value is zero (in between speckles), but a Gamma distribution that shows it is unlikely that zero intensity from one mode matches up with zero intensity from other modes. This reduction in speckle contrast must be taken into account when estimating the zero level of the structured diffraction. One way to achieve this is to fit the expected distribution to histograms of the measured intensities. More conveniently, it is possible to solve for the means of the signal and background from the moments of the measured intensities using the formulae in Table 1 ▸.

Perhaps one of the surprising aspects of the analysis is that continuous diffraction accounts for the majority of the diffracted signal. With the ability to partition photon counts into Bragg peaks, molecular diffraction and background, we found that the continuous molecular diffraction is about four times as strong as the Bragg diffraction. This is due to the greater area of diffraction space that continuous diffraction covers, compared with the Bragg peaks that only extend to a resolution of about 5 Å. The molecules in the crystal scatter the same number of photons whether those molecules are perfectly registered on a lattice or randomly displaced, and thus the diffraction counts beyond the cut-off of the Bragg peaks should be similar to the case of a perfect crystal. In a large ensemble of patterns, the total number of counts in the continuous diffraction is found to be very strongly correlated with the Bragg count, showing that the strength of both the continuous and Bragg diffractions depends on the size of the crystal. Of course, since those counts are not concentrated into sparse Bragg peaks, the signal-to-background ratio of the continuous diffraction is lower than that for Bragg peaks. For example, if individual Bragg peaks fitted into a single pixel and were spaced on average by 10 pixels in each direction, then the counts per pixel in the continuous diffraction would be about 1% of the equivalent Bragg signal. Nevertheless, since individual speckles cover similar areas to the spacings between Bragg peaks (depending on the size of the rigid unit compared with the width of the unit cell), the total count per speckle is similar to the Bragg count and the signal-to-background ratio is found to be almost comparable to the Bragg signal.

The low number of photons per pixel in continuous diffraction is of course one reason why less attention is paid to it than to the easily measured Bragg peaks. This also demands a proper treatment of counting statistics when estimating the contributions due to signal and background. For example, in the case of a continuous random variable the structureless background is considered to follow a normal distribution. This distribution has no skew, and thus any skew in the distribution of measured intensities is a signature of a structured diffraction signal. However, the Poisson distribution is skewed because there are no negative photon counts, and the skew is significant even at signal levels approaching 100 counts. It was found that only the use of discrete statistics gave rise to a reasonable estimate of the background of individual snapshot patterns, whereas non-discrete statistics resulted in over-estimation of the structured diffraction signal, as clearly illustrated in Figs. 6 ▸ and 7 ▸. The counting statistics are obviously modified by any scaling of the measured intensities, such as correction for the effect of linear X-ray polarization. In the Poisson distribution the variance is equal to the mean, which is not the case if the counts are multiplied by some factor. Regions of near-equal counts from which to determine the moments of the photon-counting distribution were therefore obtained by averaging the polarization-corrected pattern in shells of *q* and then reapplying the polarization factor. Such an approach will not be valid for background due to fluorescence or which is non-uniformly distributed across the detector face: the conditions of the experiment must be carefully controlled to avoid such contamination.

We are currently exploring the effectiveness of the approach presented here on continuous diffraction data recorded from other samples and with different types of detector. Until then, it is premature to release software, but we list in Appendix *B* [App appb] the steps of the procedure used here to obtain the three-dimensional array of continuous diffraction intensities such as shown in Fig. 9 ▸(*a*).

The fruit of the method presented here is clearly seen in the improved continuous diffraction intensity maps that are obtained – compare for example Figs. 9 ▸(*a*) and 9 ▸(*b*). The backgrounds subtracted from the individual patterns and from the merged three-dimensional array are all smooth functions that are rotationally symmetric (apart from the polarization factor). Hence the manipulations do nothing to the speckles other than locally alter their contrast. The result shows a very high degree of correlation with the continuous diffraction calculated from an atomic model, with an overall value of CC = 0.77, compared with a value of 0.55 reported previously (Ayyer *et al.*, 2016 ▸). The signal is well distinguished from background, even in the case of a strong background that was more than 25 times the diffraction signal. Stronger diffraction should arise from larger crystals, and it is worth exploring the quality of continuous diffraction with crystal size. Our first demonstration of macromolecular diffractive imaging may have been on the most challenging samples, but there may be an advantage to collecting diffraction from small volumes. Given the relative strengths of signal and background, the volume of each crystal was about 4% on average of the total probed volume of the jet, assuming the background is due entirely to the jet [and not to pulse-induced disorder of the molecules (Barty *et al.*, 2012 ▸) or conformational variability (Maia *et al.*, 2009 ▸), for example]. The maximum jet diameter was about 5 µm and the beam diameter was 1–2 µm, giving a total probed volume of up to about 20 µm^3^. Thus, the diffracting crystal volume was on average less than 1 µm^3^, even though the crystals were visually more than ten times this volume.

The analysis presented here may prove useful to studies of protein dynamics, which have examined continuous diffraction from crystals due to various kinds of differences of the constituent molecules from the average (Doucet & Benoit, 1987 ▸; Wall *et al.*, 1997 ▸; Pérez *et al.*, 1996 ▸; Van Benschoten *et al.*, 2016 ▸). Such measurements are usually not time resolved and hence cannot distinguish static from dynamic disorder, but these measurements can be compared with diffraction calculations based on molecular dynamics trajectories to gain, it is hoped, insights into protein motion and function. As shown here and in the previous work (Ayyer *et al.*, 2016 ▸), one can establish the origin of continuous diffraction and account for dominant effects (such as translation of rigid units) prior to examining the effects of correlated motions. For example, the autocorrelation function obtained by a Fourier transform of the continuous diffraction intensities reveals the shape and size of the rigid units. Indeed, previous studies of lysozyme crystals determined from the speckle size that the rigid unit was the size of a lysozyme molecule (Pérez *et al.*, 1996 ▸), and considerations of the mechanical properties of protein crystals led to the conclusion that molecular translations and rotations (dependent on the elastic and shear moduli, respectively) in these bodies are inevitable (Morozov & Morozova, 1986 ▸). Based on the formalism of Morozov & Morozova (1986 ▸), a molecular displacement of σ_Δ_ = 2 Å at room temperature for a molecular width of 178 Å implies a value of Young’s modulus of only 0.01 GPa. This is similar to soft rubber, which is consistent with experience in handling these crystals.

The statistics of diffraction intensities, and in particular the speckle contrast, directly yield information on the number of independent modes contributing to the diffraction. Together with the symmetry of the crystal, this can indicate whether such independent objects correspond to asymmetric units in the crystal, but we stress that the methodology of our approach may need to be modified to account for various types of disorder that can occur in crystals or for conditions other than the random and isotropic translational disorder considered here. Comparing centric and acentric sections gives further evidence for the origin of the diffraction. In all studies of continuous diffraction it is imperative to measure the diffraction accurately at a sufficient sampling in all three dimensions, and to remove the background that accompanies such measurements. The approach given here is shown to be effective in extracting single-molecule diffraction from patterns arising from translationally disordered crystals and it is expected that it may similarly improve measurement of continuous diffraction due to other kinds of disorder.

Although beyond the scope of this paper, we expect that the improved treatment of the background should lead to better structure determination from continuous diffraction through iterative phasing. A better correlation with simulated diffraction was found by assuming a 1° rotational disorder of the PS II dimers. For the 178 Å diameter dimer, this will cause a blurring of the structure at resolutions beyond 3 Å, but by using methods of partially coherent diffractive imaging (Whitehead *et al.*, 2009 ▸) it should be possible to take such blurring into account, to a resolution where speckles are no longer visible. That the speckles do indeed appear visible to the edge of the detector at 2 Å resolution suggests that, with more measurements, structural information should be obtainable to at least that resolution.

## Figures and Tables

**Table 1:** Moments of the distribution of intensities obeying noisy Wilson statistics

	*p*_NW_(*I*; *N*)	*p*_NW_(*I*; *N* − 1, 1)	*p*_DNW_(*I*; *N*)
Mean (μ_NW_)	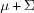	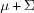	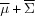
Variance ( 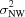 )	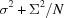	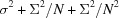	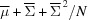
Skewness (*s*_NW_)	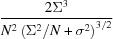	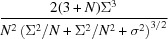	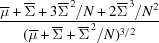

## Appendix A The ‘noisy Wilson’ distribution

We consider non-discrete acentric diffraction intensities incoherently summed with a background *I*_B_ that is normally distributed with a mean μ and variance σ^2^, *I*_B_ ∼ , with

We refer to the distribution of this sum *I* ∼ Gamma(*N*, Σ/*N*) +  as the noisy Wilson distribution, with a probability distribution function found by convolution of equations (3) and (15) as

where

and  is the confluent hypergeometric function. For *N* = 1, 2, 4, 6 and 8,  evaluates to

where  is proportional to the scaled complementary error function,

Some plots of *p*_NW_(*I*) are given in Figs. 2 ▸(*c*) and 2 ▸(*d*).

Equations (16) and (17) can be evaluated for non-integer values of *N*, such as those needed to account for partial coherence, by computing the series expansion of the confluent hypergeometric function.

## Appendix B Procedure for processing diffraction data

The following itemizes the steps taken to process still diffraction patterns of crystals recorded in random orientations or a series of orientations. Here, it is assumed that the patterns are recorded with a common (unchanging) detector geometry and wavelength, the incident beam is linearly polarized, and the unstructured background is radially symmetric when corrected by the polarization factor. The procedure also assumes intensity data in units of photon counts, obtained using either a well calibrated integrating detector or a photon-counting detector.

(i) Process the data set using *CrystFEL* (White *et al.*, 2012 ▸, 2016 ▸) to find the indexable patterns and the lattice orientation for each indexed pattern, as well as to create a set of merged Bragg intensities.

(ii) For each indexed pattern (calibrated and bad areas of the detector masked, but otherwise uncorrected for polarization):

(*a*) Mask Bragg peaks using a threshold filter or the *peakfinder8* procedure from *Cheetah* (Barty *et al.*, 2014 ▸). Dilate the mask by a kernel about 7 pixels wide. The intensities in the masked pixels are not included in any further analysis.

(*b*) Create contours of *I*_av_ [equation (7)] at a spacing of about 1 photon count, and from these determine contiguous regions bounded by those contours.

(*c*) Calculate the moments (mean and variance) of photon counts in each contiguous region to compute the parameters of the discrete noisy Wilson distribution ,  from equations (14) with the appropriate value of *N* (here *N* = 4).

(*d*) Set the background  to values  for each region, then smooth with a square kernel about 3 pixels wide.

(*e*) Determine a scaling array  in a similar way to Step (ii*d*).

(*f*) Subtract the smoothed background of Step (ii*d*) from the pattern.

(*g*) Normalize the pattern by dividing by the sum of  from Step (ii*e*) over a predetermined range of |**k**|.

(*h*) Correct for polarization by dividing by the polarization factor  [equation (7)].

(iii) Merge the corrected patterns into a three-dimensional reciprocal-space volume by interpolating each onto the appropriate Ewald sphere in the frame of reference of the crystal lattice (Yefanov *et al.*, 2014 ▸). The spacing of voxels in the three-dimensional array should be chosen to sample sufficiently the highest frequencies of the continuous diffraction, Δ*q*  1/(2*w*), where *w* is the width of the rigid body.

(iv) Apply the symmetry operations of the point group of the diffraction to the three-dimensional array (here, point group 222).

(v) Calculate the moments of the scaled intensities in shells of *q* from the three-dimensional array and calculate the parameters of the noisy Wilson distribution μ, σ and Σ in those shells *via* equations (13), avoiding centric zones.

(vi) Construct μ(**q**) for all voxels of the three-dimensional array, smooth it with a three-dimensional kernel about 3 pixels wide and then subtract this from the merged intensities, to account for residual background.

(vii) Determine the scaling and background of the merged Bragg intensities [from Step (i)] in shells of *q* by applying the same procedure as in Step (v) but with the appropriate value of *N* (here *N* = 1). Fit the Debye–Waller factor to the values Σ(*q*) for these Bragg intensities to obtain σ_Δ_.

(viii) Correct the continuous diffraction intensities by dividing by the complementary Debye–Waller factor .
